# The PRP19 Ubiquitin Ligase, Standing at the Cross-Roads of mRNA Processing and Genome Stability

**DOI:** 10.3390/cancers14040878

**Published:** 2022-02-10

**Authors:** Mouhamed Idrissou, Alexandre Maréchal

**Affiliations:** 1 Faculty of Sciences, Department of Biology, Université de Sherbrooke, Sherbrooke, QC J1K 2R1, Canada; mouhamed.idrissou@usherbrooke.ca; 2 Centre de Recherche du Centre Hospitalier Universitaire de Sherbrooke, Sherbrooke, QC J1H 5N3, Canada

**Keywords:** mRNA processing and splicing, ubiquitin ligase, DNA damage response, genome stability, RNA:DNA hybrids

## Abstract

**Simple Summary:**

mRNA maturation is absolutely required for proper gene expression and, in recent years, regulators of this process have been found to be tightly intertwined with genome stability. The E3 ubiquitin ligase PRP19 is part of multiple protein complexes that regulate mRNA splicing, RNA:DNA hybrid resolution, activation of the ATR-mediated DNA damage response, DNA repair and cell division. Here, we discuss how this essential evolutionarily conserved factor functions at the nexus between mRNA processing and genome protection and we highlight key questions that will need to be addressed to better understand the interface between gene expression and genome stability.

**Abstract:**

mRNA processing factors are increasingly being recognized as important regulators of genome stability. By preventing and resolving RNA:DNA hybrids that form co-transcriptionally, these proteins help avoid replication–transcription conflicts and thus contribute to genome stability through their normal function in RNA maturation. Some of these factors also have direct roles in the activation of the DNA damage response and in DNA repair. One of the most intriguing cases is that of PRP19, an evolutionarily conserved essential E3 ubiquitin ligase that promotes mRNA splicing, but also participates directly in ATR activation, double-strand break resection and mitosis. Here, we review historical and recent work on PRP19 and its associated proteins, highlighting their multifarious cellular functions as central regulators of spliceosome activity, R-loop homeostasis, DNA damage signaling and repair and cell division. Finally, we discuss open questions that are bound to shed further light on the functions of PRP19-containing complexes in both normal and cancer cells.

## 1. RNA Processing and DNA Repair, a Long-Standing Partnership for Genome Stability

In order to maintain genomic stability, cells have to deal with a wide variety of lesions and obstacles to DNA replication created by endogenous and exogenous stress. These situations are effectively handled by a collection of signaling pathways termed the DNA damage response (DDR) that repair DNA lesions, enable faithful replication and preserve genomic information [1,2,3].

In recent years, RNA:DNA hybrids formed as a consequence of transcription–replication conflicts and ribonucleotide insertions have emerged as prevalent threats to genome integrity. In particular, three stranded structures known as R-loops containing an RNA:DNA hybrid and an extruded single-stranded DNA fragment have been recognized as important regulators of gene expression that normally accumulate within gene promoters and at repetitive sequences such as transposons, telomeres and centromeres. Increasingly, R-loops have also been found to play positive roles in genome stability, for instance by promoting telomere and double-strand break (DSB) repair (reviewed in [4]). Despite these central cellular functions, unscheduled R-loop formation is now recognized as an important source of DNA replication stress and DNA damage. Work from many labs performed in model organisms ranging from bacteria to yeast and human cells has produced a far-from-complete picture in which genome maintenance and RNA maturation factors collaborate to prevent and resolve RNA-containing structures that can disrupt genome replication (reviewed in [5,6,7]). Highlighting the far reaching consequences of disrupting the homeostasis of these structures, defects in co-transcriptional RNA processing and/or stabilization of R-loops have been linked to cancer, neurodegenerative triplet repeat expansion diseases, Aicardi–Goutières inflammatory encephalopathy, Fanconi anemia and a number of other debilitating syndromes ([8,9] and reviewed in [10]).

Both passive and active roles have been proposed for a host of DDR and RNA processing proteins in the mitigation of RNA-induced genome instability. For instance, impaired mRNA splicing and packaging which normally occurs co-transcriptionally in eukaryotes leads to profound genome destabilization [11,12]. A number of RNA helicases actively unwind RNA:DNA hybrids, specialized RNAses (e.g., RNAseH1/2) can remove incorporated ribonucleotides or R-loops from the genome and endonucleases (e.g., XPF, XPG) can recognize and cleave R-loops in cells [13,14,15,16,17]. The DDR can also sense and minimize the genotoxic effects of R-loops. Head-on collisions between RNA polymerase II and the replisome in an engineered episomal system were shown to activate the master ATR kinase while co-directional conflicts led to ATM activation [18]. Splicing defects or inhibition of the RNA:DNA hybrid helicase senataxin activate ATR during S-phase which in turn protects against R-loop-mediated genome destabilization [19]. Similarly, ATR inhibition in synovial sarcoma cells led to enhanced R-loop formation and DNA damage that correlated with sensitivity to ATRi [20]. Increased R-loop formation induced by myelodysplastic syndrome-associated mutations in the spliceosome proteins U2AF35 and SRSF2 was also shown to activate the ATR but not the ATM branch of the DDR and U2AF35(S34F)-expressing cells were sensitive to ATR inhibition [21,22]. Depleting ATR, ATM or their downstream kinases CHK1 and CHK2 all lead to R-loop accumulation in human cells cementing the importance of these central signaling pathways in protecting against RNA:DNA hybrids [23]. Downstream of R-loop detection, ATR and ATM protect against RNA-related genotoxicity in various ways. For instance, ATR and ATM can promote recruitment of senataxin to sites of DNA replication stress and ATR activation promotes nuclear import of RNA helicase DDX19 to decrease R-loop levels [24,25]. ATR activation in response to splicing inhibition requires replication fork reversal and processing by the structure-specific nuclease MUS81. Once activated, ATR decreases transcription-replication conflicts and MUS81-mediated double-strand breaks (DSBs) while also stopping progression into mitosis [19]. Thus, cells possess a plethora of mechanisms to prevent the formation of RNA-containing structures and can also rely on dedicated sensors, mediators and effectors to evade the genome destabilizing consequences of unscheduled R-loops. One of the most interesting cases of a factor acting at the RNA–genome stability interface is the E3 ubiquitin ligase PRP19 and its associated complexes which play both direct and indirect roles upstream and downstream of RNA processing to protect genome stability. Here, we summarize the historical and more recent discoveries that have positioned this intriguing ubiquitin ligase at the nexus of DNA and RNA metabolism.

## 2. PRP19/PSO4, an RNA Processing Factor and DNA Damage Response Regulator

Pre-mRNA Processing factor 19 (PRP19) was first implicated in RNA maturation by a yeast forward genetics screen designed to identify genes involved pre-mRNA splicing. In total, ~1000 thermo-sensitive *Saccharomyces cerevisiae* strains were screened for intron-containing actin transcripts at the non-permissive temperature leading to the identification of 11 new RNA processing complementation groups [26]. The *prp19-1* mutant accumulated actin and RP51A pre-mRNA, indicative of a function in the early steps of mRNA splicing. Further studies from the Abelson lab led to the cloning of *PRP19*, an essential gene shown to encode a spliceosome-associated protein required for the first cleavage-ligation reaction at introns [27]. Alterations in *PRP19/PSO4* also lead to sensitivity to multiple DNA damaging agents. In fact, the first *PRP19/PSO4* mutant (*xs9*) was initially isolated in the 1970s during an X-ray sensitivity screen and later found to confer extreme susceptibility to 8-methoxypsoralen photoaddition (8-MOP + UV-A) which predominantly produces interstrand DNA crosslinks (ICLs). Accordingly, *xs9* was renamed *pso4-1* as it was non-allelic to the other three psoralen-sensitive strains described at the time [28,29]. *pso4-1* is also sensitive to UV, nitrogen mustard and methyl methane sulfonate, is hypomutable when exposed to these genotoxins and has impaired mitotic recombination (gene conversion and crossing over) [30,31]. Further mechanistic studies showed that incision at psoralen-induced crosslinks occurred normally in *pso4-1* mutants but the recombination-mediated rejoining step was impaired. Moreover, *PSO4* was epistatic with *RAD51* and *RAD52* with respect to psoralen and MMS sensitivity, therefore positioning *PSO4* in the *RAD52* complementation group of DNA repair genes [32]. Again, the spontaneous hypermutation induced by *RAD51* and *RAD52* mutations was muted in *pso4-1* double mutants indicating that *PSO4* is involved in a recombination-based error prone pathway. Molecular cloning of *PSO4* revealed its allelism to *PRP19*, confirmed its essentiality for cell survival and identified it as the first protein with dual roles in mRNA processing and DNA repair. The *pso4-1* mutant strain contains an L45S mutation in the U-box domain of the protein likely impacting its E3 ubiquitin ligase activity and perhaps its ability to self-associate [33,34,35]. The fact that no other splicing factor was linked to DNA repair via classical genetics supported the idea that PRP19/PSO4 plays a dual function in mRNA processing and the DNA damage response. Moreover, only mild defects in splicing were observed at permissive temperatures, yet strong sensitivity to DNA damaging agents was found at all temperatures examined for *pso4-1* strains, reinforcing the idea that PRP19/PSO4 is a multifunctional essential protein with critical roles in RNA processing and the DDR [36].

## 3. Molecular Architecture of PRP19 Complexes

**Figure 1:**
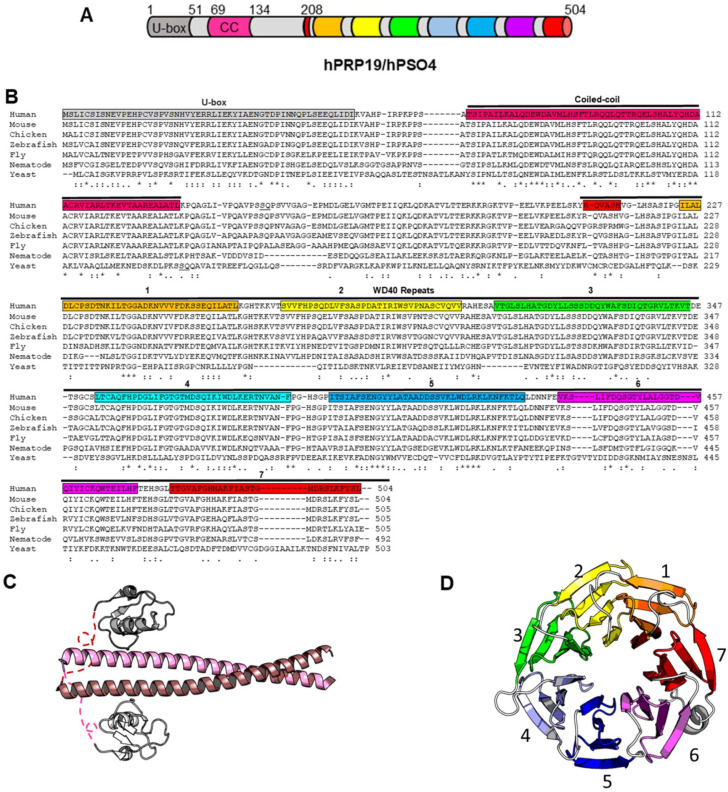
**PRP19 structural domains.** (**A**) Schematic representation of hPRP19. (**B**) PRP19 is a highly conserved U-box E3 ubiquitin ligase in eukaryotes. Protein sequences of PRP19 homologs were obtained from the Uniprot database and aligned using Clustal Omega. Boxshade was used for alignment formatting. The (*), (:) and (.) symbols denote identical, conservative and semi-conservative amino acid positions, respectively. (**C**) Crystal structure of the stalk domain of *Chaetomium thermophilum* PRP19 (PDB 5M88) in dimeric form comprises U-box domains in grey and coiled-coil regions in pink/salmon. In this conformation, the U-box domains interact with the coiled-coil regions of the opposite protomers, rendering the α-helix and adjacent loops that normally interact with E2-conjugating enzymes unavailable. (**D**) Crystal structure of the WD40 repeat substrate-binding domain of human PRP19 (PDB 4LG8).

PRP19 is a highly conserved E3 ubiquitin ligase comprising a U-box domain, a coiled-coil tetramerization domain and a 7-bladed WD40-repeat substrate binding domain (Figure 1A–D) [35,37,38,39,40,41]. The U-box fold is similar to that of the RING E3 ligase domains with the key difference that the zinc ions that normally promote the proper folding of RING domains are replaced by a network of hydrogen bonds and salt bridges [35]. The U-box and coiled-coil domains form a stalk-like structure and when PRP19 self-assembles as an homotetramer composed of two colinear dimers, the U-box surfaces that normally contact E2 conjugating enzymes interact with the opposing coiled-coil domains of their dimeric partners, effectively quelling the E3 ubiquitin ligase activity of PRP19 (Figure 1C).

Mutation of the conserved coiled-coil residues that interact with the U-box domains can artificially release this inhibition. Autoinhibition of PRP19 is also relieved when it associates with CDC5L, SPF27 and PLRG1 to form the active nineteen core complex (NTC) with the PLRG1 subunit being essential for this activation. All four core subunits of the complex are co-dependent for stability in vivo underlining their tight physical and functional association. Upon PRP19 activation, the four U-box domains form two active dimers that can contact E2s loaded with ubiquitin and transfer ubiquitin chains onto substrates [34]. Mutation of the dimer interface residues of the U-box is lethal at non-permissive temperature in vivo, demonstrating the importance of this architecture for PRP19 function. Three additional subunits (CTNNBL1, AD-002/CWC15 and HSP73) also associate less tightly with the NTC in human cells, forming the complete active PRP19 complex [42]. CTNNBL1 is an ARM domain protein with structural features reminiscent of karyopherins. As such, it has been found to bind nuclear localization signals on various factors and influence their subcellular distribution (e.g., CDC5L, PRP31 and activation-induced cytidine deaminase involved in antibody diversification [43,44,45]). CTNNBL1 was shown to be required to maintain normal levels of the PRP19 complex and enhance the association between CDC5L and AD-002/CWC15. Moreover, the more labile nature of the association of CTNNBL1 and AD-002/CWC15 with CDC5L and the rest of the NTC suggest a dynamic exchange of these two proteins within the PRP19 core complex [46]. CTNNBL1 was thus proposed to function as a chaperone for the NTC, enhancing its functions in mRNA splicing and the DNA damage response.

**Figure 2:**
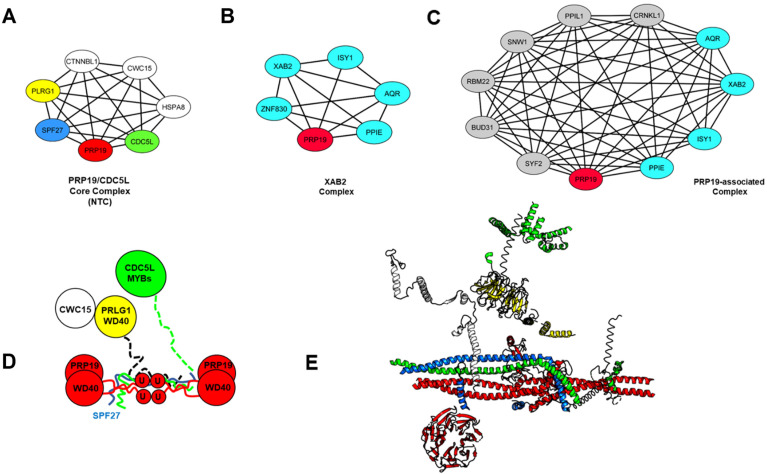
**Composition and architecture of human PRP19-containing complexes.** (**A**–**C**) Network analysis of PRP19 complexes. Interactome data for the three human PRP19-containing complexes was obtained from the STRING database using the highest confidence settings and processed using Cytoscape. Edges correspond to experimentally validated protein–protein interactions. (**D**) Schematic representation of the architecture of the PRP19/CDC5L (NTC) core complex. The tetrameric coiled-coil of PRP19 forms the core of the complex with the WD40 substrate binding domains radiating outward and two dimeric U-box assembling in the center of the complex. The PRP19 coiled-coil also interacts with the alpha-helical domains of CDC5L, PLRG1 and SPF27. (**E**) Cryo-EM structure of the PRP19 core complex from *Schizosaccharomyces pombe* (PBD:3JB9) within the spliceosome. Only the structures of the PRP19 core complex proteins visible within the spliceosome are shown for simplicity. PRP19: red; CDC5L: green; SPF27: blue; PLRG1: yellow; CWC15: white.

Crosslinking, two-hybrid genetics, biochemical and structural studies have revealed the overall architecture of the heptameric PRP19 complex. CDC5L and SPF27 interact with the tetrameric coiled-coil domain of PRP19 via alpha-helical domains at their C- and N-terminal portions, respectively [37,40]. In the activated conformation, two dimeric U-box domains are found above the central portion of the PRP19 tetramerization domain (Figure 2A,D,E).

**Figure 3:**
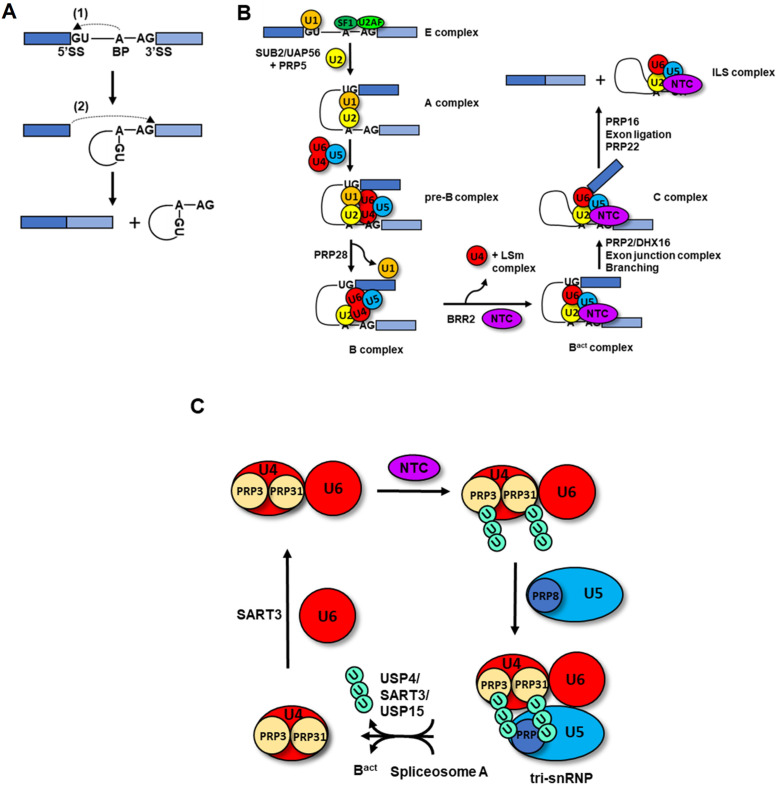
**The PRP19 complex in the splicing cycle**. (**A**,**B**) The spliceosome is a highly dynamic complex that catalyzes two transesterification reactions to remove introns and relegate exons together (see text for further details). (**C**) A ubiquitylation–deubiquitylation cycle regulates tri-snRNP formation and recycling. The NTC complex ubiquitylates U4 proteins PRP3 and PRP31 with K63-linked ubiquitin chains. These chains associate with the JAMM-MPN1 domain of the PRP8 core U5 snRNP factor reinforcing the stability of the tri-snRNP complex. The tri-snRNP complex enters the spliceosome at the A to B transition. During the B to B^act^ maturation step, USP4/SART3/USP15 promote deubiquitylation of PRP3 and PRP31 destabilizing the U4-U5 association and potentially helping spliceosome maturation. The free U4 snRNP reassociates with U6 with the help of SART3 and can re-enter the splicing cycle upon NTC-mediated ubiquitylation.

In addition to the NTC complex, PRP19 is part of at least two other multiprotein complexes in human cells [47], (Figure 2B,C). Isolation of interacting partners of the multifunctional XAB2/SYF1 (XPA-binding protein 2/Synthetic lethal with CDC40 protein 1) tetratricopeptide repeats protein in HeLa cells revealed the existence of a hexameric complex containing PRP19, XAB2, ISY1, ZNF280E, PPIE and the RNA helicase AQR with functions in nucleotide excision repair (NER), transcription and mRNA splicing. Depletion experiments also showed that XAB2 depletion destabilized AQR and ISY1 but that conversely, PRP19 depletion had no effect on the levels of the other members of the XAB2 complex [48]. The PRP19-associated complex has a more heterogeneous composition and was mostly defined by proteomics studies of yeast and human post-activation spliceosomes and via tandem-affinity purifications of Cdc5/Cef1/CDC5L-associated proteins in budding and fission yeasts. It is composed of at least 26 proteins in yeast and includes the NTC along with other core spliceosome factors. This complex becomes associated with the spliceosome at the transition between the pre-catalytic (B) and the activated (B^act^) spliceosome prior to the first esterification reaction (Figure 3) [49,50,51].

## 4. Roles of PRP19-Containing Complexes in RNA Maturation

Most mammalian pre-mRNAs contain exons and introns. These immature mRNAs must be processed by removing introns and ligating exons to produce mRNAs with continuous protein-coding sequences. Alternative splicing generates multiple mRNA isoforms from single genes greatly enhancing the overall coding capacity and flexibility of eukaryotic genomes. Splicing occurs primarily co-transcriptionally as pre-mRNAs exit RNA pol II and this process is critical for eukaryotic cells. Tellingly, most core splicing factors in human cells, including NTC components, were identified as essential factors by recent genome-wide CRISPR-Cas9-based fitness screens, emphasizing the importance of proper RNA maturation for cell viability [52]. Furthermore, mutations in splice sites or splicing factors have been causally implicated in a wide variety of diseases including cancer and neurodegenerative disorders [53].

mRNA splicing is catalyzed by the spliceosome, a large and dynamic ribonucleoprotein machine that undergoes several cyclic conformational changes to achieve precise mRNA maturation [54]. The spliceosome is composed of five snRNPs and several non-snRNP proteins that interact dynamically during a single splicing reaction. Recent cryo-electron microscopy studies of yeast and subsequently mammalian spliceosomes at various splicing steps have provided detailed structural information on the key transitions that enable co-transcriptional mRNA maturation [55,56,57,58,59,60,61,62,63,64]. Here, we provide a brief description of the major conformational changes that underlie spliceosome function (Figure 3A,B), centering on the roles of the PRP19 complex (Figure 3C) in this process, and refer readers to excellent recent review articles on the structural dynamics of the spliceosome for more details [65,66].

As depicted in Figure 3A, the splicing reaction entails two transesterifications that occur (1) between the 2′OH of the branch point A and the 5′ phosphate of the intron and (2) between the newly formed 3′OH of the first exon and the 5′ phosphate of the 3′ splice site producing joined exons and an intron lariat. In the early steps of splicing, U1 snRNP pairs with the 5′ splice site (5′SS) to form the E complex (Figure 3B). Simultaneously, the branch point (CP) adenosine, an adjacent pyrimidine tract and the 3′ splice site are recognized by SF1/SRSF2 and the U2AF complex. Then, the U2 snRNP with the help of RNA helicases UAP56 and PRP5/DDX46 recognizes the branch point to form the A complex. A pre-formed tri-snRNP U4/U6.U5 containing the PRP28 RNA helicase then associates with the A complex to form the pre-B complex. PRP28 RNA helicase promotes remodeling of U1 snRNP and its replacement by U6 snRNP which now contacts the 5′SS in the B complex. The BRR2 helicase unwinds U4/U6 interactions allowing U6/U2 pairing and enabling transition into the catalytically active B^act^ complex. During this transition, the PRP19-associated complex and the NTC also engages the spliceosome after U4 departure while the Lsm complex is also removed. The PRP2/DHX16 helicase promotes extensive remodeling of the B^act^ complex into the B* complex that catalyzes the first transesterification reaction. Addition of the exon junction complex leads to C complex formation and after branching, the PRP16 helicase rearranges the C complex into C* in which the exon ligation step occurs. The PRP22 ATPase releases the ligated exons and the intron lariat spliceosome complex (ILS).

## 5. Roles of the NTC and PRP19-Associated Complex in RNA Splicing

Proteomics, crystallographic and electron microscopy data have shown that the NTC and components of the PRP19-associated complex interact dynamically with the spliceosome at various steps of the splicing cycle. U2AF65 was shown to recruit the PRP19 complex to the pre-mRNA by acting as a tether to the phosphorylated C-terminal domain of RNA pol II and this may help the NTC integrate activated spliceosomes in a co-transcriptional manner [67]. This strategy for the recruitment of the NTC to elongating RNA pol II appears to be evolutionarily conserved as it is also shared by Mud2, the putative *S. cerevisiae* homolog of U2AF65 [68]. NTC recruitment occurs at the B^act^ step of the spliceosome cycle typically after U4 snRNP displacement as determined by single-molecule spliceosome maturation studies and by proteomics analysis of B spliceosomes blocked at an intermediate stage prior to B^act^ [69,70,71]. Consistent with its association with the spliceosome from the B^act^ until the intron lariat (ILS) post-catalytic form, yeast *PRP19* mutation impairs the first cleavage–ligation reaction as precursor mRNA accumulates at the non-permissive temperature [27,72]. Moreover, immunodepletion of the NTC using CDC5L and AD-002-specific antibodies inhibited the first catalytic step of splicing in human cell extracts [73]. Elegant yeast splicing extracts experiments further showed that the NTC stabilizes the binding of U5 and U6 on the spliceosome but is not involved in U4 dissociation during the B to B^act^ spliceosome transition. UV crosslinking experiments also indicated that the NTC promotes base pairing shifts between U6 snRNA and the 5′ splice site and also influences association of the 3′ end of U6 snRNA with the intron sequence adjacent to the 5′SS. Moreover, association of the LSm complex with the spliceosome was enhanced in NTC-depleted yeast extracts, suggesting that Prp19 promotes eviction of these factors during spliceosome activation [74]. Further crosslinking studies indicated that the NTC may help specify base-pairing interactions between U5, U6 and pre-mRNA in the active spliceosome [75]. Synthetic lethality between mutant alleles of U6 snRNA and the Isy1 PRP19-associated complex subunit provides genetic support for a role in catalytic site architecture for PRP19 and its partners [76]. A yeast screen for mutants with defective U4 snRNA assembly into functional snRNPs also indicated that Prp19 prevents the accumulation of free U4 snRNA and thus promotes the assembly or stability of the U4/U6 particle [77]. This finding was confirmed by the Cheng lab who showed that PRP19 and NTC25/SPF27 mutation destabilized U4/U6 snRNP, increased free U4 snRNA and led to decreased levels of U6 snRNA, hinting at additional roles for the NTC in U4/U6 snRNP biogenesis and spliceosome recycling [78]. Evidence also exists for PRP19-associated complex roles after the first transesterification step of splicing. For instance, mutation of the Isy1 PRP19-associated complex subunit can rescue a cold-sensitive allele of the Prp16 DEAH-box RNA helicase which normally drives the transition between the two splicing steps [76]. Moreover, immunodepletion of the NTC in HeLa cell extracts led to strong impediment of the second transesterification whereas the first splicing step was less affected [79].

There is strong biochemical and structural evidence that the NTC directly contacts RNAs in the active site of the spliceosome in the B^act^, C and post-catalytic spliceosomes in both yeast and humans [80,81,82,83]. Indeed, the N-terminus of *S. pombe* Cdc5 is essential for cell viability and binds regions within the U2 and U6 snRNAs in vitro. Moreover, a purified protein containing both Myb and Myb-like/coiled-coil domains of Cdc5 bound more tightly to U2 and U6 ss- and dsRNA than individual domains suggesting that these motifs may interact cooperatively with multiple RNA moieties at the spliceosome catalytic center, perhaps enabling conformational transitions during spliceosome activation while the C-terminus of Cdc5 anchors it to the rest of the NTC [84]. The structure of the NTC within various states of spliceosome activation shows that the CDC5L Myb and coiled-coil N-terminal domains extend into the catalytic center of the spliceosome and indeed contacts the U6 snRNA ([64] and reviewed in [85]. Additionally, the RNA-binding component of the PRP19-associated complex RBM22/CWC2 interacts with the PRP19 WD40 domain and anchors the PRP19 complex to spliceosomal RNAs, in particular U6 snRNA via its RRM domain [39,63,85,86]. RNA–protein interactions also occur between SNW1, CRNKL1, AD-002 and PLRG1 and the active face of the U2/U6 snRNA again mostly via U6 in the B^act^ human spliceosome (reviewed in [85]). Altogether, these data strongly support a model whereby the NTC and PRP19-associated complex proteins act as chaperones to guide the structural transitions that lead to an active conformation of the spliceosomal RNA network.

PRP19 itself also participates in spliceosome remodeling via its E3 ubiquitin ligase activity. The first evidence for ubiquitylation-mediated regulation of splicing came from the discovery that a I44A ubiquitin mutant, which can be conjugated but has impaired association with ubiquitin binding domains, compromised splicing and led to reduced U4/U6.U5 snRNP levels when added to yeast extracts. In the same study, the U5 snRNP core protein Prp8 was also shown to be ubiquitylated [87]. In support for a non-degradative role of ubiquitylation in splicing regulation, the variant JAB1/MPN1 domain of Prp8p, a component of the U5 snRNP was found to directly bind ubiquitin and its mutation impaired splicing [88]. The *prp19-1* mutation lies within the conserved U-box motif of PRP19 (V14I) and disrupts the structural integrity of the domain, thereby directly implicating the E3 ubiquitin ligase activity of PRP19 in splicing regulation and the DNA damage response [34,35]. NTC-mediated ubiquitylation has emerged as a critical regulator of tri-snRNP assembly during the splicing cycle (Figure 3C). PRP19 ubiquitylates the U4 snRNP component PRP3 with non-proteolytic K63-linked ubiquitin chains increasing its association with the U5 snRNP protein PRP8 via its partial JAMM motif and stabilizing U4/U6.U5 snRNP. In support of this last point, overexpression of the USP4 deubiquitylase destabilized the tri-snRNP and U4/U6 recycling was shown to be mediated by PRP3 deubiquitylation by USP4/SART3 [89]. Follow up work from the Song lab, revealed that PRP31, a U4/U6 snRNP protein required for tri-snRNP stability in vivo, and the PRP4 kinase found in the U4 snRNP are both substrates of PRP19 [90]. K63-linked ubiquitylation of PRP31 by PRP19 regulates its association with PRP8 and the U5 snRNP. This ubiquitylation is countered by the USP15/USP4/SART3 complex to enable formation of tri-snRNP particles and perhaps modulate remodeling of the U4/U6.U5 complex during spliceosome activation [90]. In accordance with this model, overexpression of USP15 and USP4 destabilized the association of PRP31 with the PRP8 JAMM domain while increasing PRP19 levels enhanced it. Thus, in addition to a direct role in active site architecture regulation, the NTC regulates tri-snRNP formation and U4/U6 recycling within a ubiquitylation–deubiquitylation cycle.

## 6. Regulation of Gene Expression, Cell Fate and Development by NTC and PRP19-Associated Complexes

Curiously, the impact of NTC depletion or mutation on mRNA expression and splicing patterns has thus far been very sparsely studied. In human normal fibroblasts, it was recently found that PRP19 is downregulated during replicative senescence and that its depletion (KD) induces the p53-p21 cell cycle checkpoint pathway [91]. PRP19 KD also induced spontaneous accumulation of DNA damage as previously shown but depletion of ATR or ATM did not alter p53 and p21 accumulation in PRP19 KD cells [12]. Moreover, p53 mRNA maturation and expression was not altered by PRP19 depletion. Global RNA expression profiling revealed that PRP19 KD alters ~2000 mRNA splicing events, mostly inducing exon skipping. More specifically, skipping of exon 6 in the negative p53 regulator MDM4 was found to promote accumulation of an unstable MDM4-S isoform that accelerated senescence in primary diploid cells. Overexpression of MDM4-FL attenuated activation of the p53-p21 pathway in PRP19 KD cells supporting a model in which impairment of MDM4 splicing by PRP19 KD promotes senescence in PRP19-depleted cells. Downregulation of PRP3 and PRP8 also impaired MDM4 splicing, in line with the role of PRP19 in promoting association of U4 and U5 snRNPs during the splicing cycle. These results contrast with prior work from the Jones lab that showed that KD of PRP19 itself or of the PRP19-associated protein SNW1/SKIP induced p53 expression but impaired p21 expression and splicing without affecting induction of the pro-apoptotic p53 targets PUMA and NOXA during genotoxic stress. This ultimately led to enhanced DNA damage-induced apoptosis upon SNW1 depletion. One possible explanation for this discrepancy is the use of DNA damage-inducing agents and cancer cell lines in one study as opposed to senescent normal fibroblasts in the other. It is likely that splicing regulation by PRP19 and its associated factors varies depending on cell types and growth conditions and further studies are required to fully grasp the effect of NTC and PRP19-associated factors depletion on gene expression programs [92].

In *Ustilago maydis*, deletion of the NTC subunit Num1/SPF27 produced viable cells with impaired plant infection capabilities and aberrant filamentous growth. Δnum1 cells were also vulnerable to genotoxic stress (UV, phleomycin and hydroxyurea). RNA-Seq experiments showed that Δnum1 cells had a global alteration of the splicing pattern. Intron retention was particularly prevalent and twice as high as in WT strains but no specific gene ontology enrichment could be detected for genes with high intron retention rates [93].

In drosophila, development of the embryo begins by a rapid series of nuclear divisions in the fertilized egg while the cell itself does not split. Fly embryos still need to express many genes despite undergoing rapid successive mitoses which repress gene expression. Thus, maternally inherited mRNAs are used at first prior to the embryo making its first mRNAs. The extremely rapid divisions that follow zygote formation limit the time that can be spent on transcription and splicing of genes and thus a selection seems to have occurred to favor short and intronless early embryonic mRNAs. This developmental step also requires highly efficient splicing machineries since *XAB2/Fandango* hypomorphic mutants failed to splice out introns of early embryonic mRNAs but correctly spliced maternally inherited mRNAs. A stronger non-sense *fandango* mutation led to complete loss of the female germline in adult ovaries indicating that a lower level of Fandango is required for splicing of maternal transcripts [94]. Similarly, mutation of *Salsa*, the drosophila homolog of *Aquarius*, a PRP19-associated RNA helicase, also impairs splicing of a subset of small first introns during oogenesis [95]. Altogether, this data indicates that splicing efficiency requirements vary during development and that highly proliferative tissues need to coordinate cell cycle progression and gene architecture to avoid RNA transcription and maturation issues.

In addition to its role as a central regulator of RNA splicing, the PRP19-associated complex also participates in transcriptional elongation and mRNA export from the nucleus [47]. In *S. cerevisiae*, a synthetic lethal relationship was established between SYF1/XAB2 and the TREX complex which couples transcription and mRNA export [96]. An RNA-independent interaction was also found between Syf1 and Hpr1, a THO subunit of TREX. ChIP analysis showed that Syf1 and Prp19 associate with intronless genes as well as intron-containing genes. A shift to the non-permissive temperature of *syf1-37* cells led to a decrease in transcriptional activity that could be complemented by adding back the functional PRP19-associated complex. Syf1 was required for recruitment of the PRP19-associated complex to transcribed genes and the association of Hpr1, Sub2 and Yra1 with the 3′ end of actively transcribed genes decreased by ~50% in *syf1-37* cells demonstrating its role in TREX complex recruitment. The function of the NTC and associated proteins in mRNA export appears to be conserved in humans where PRP19, CDC5L, AQR, XAB2, U2AF65 and components of the TREX complex were found to bind to cytoplasmic accumulation regions found in naturally intronless transcripts. Depletion of XAB2, CRNKL1 and ISY1 led to nuclear retention of IFNβ1 intronless RNAs. However, depletion of PRP19 itself, PPIE and PLRG1 did not affect nuclear retention of these transcripts suggesting that certain components of the NTC-associated complexes might play more prominent roles in this process or that depletion efficiency was insufficient to reveal a phenotype. Further evidence for PRP19-associated complex roles in mRNA maturation and export comes from a recent genome-wide CRISPR screen for factors repressing the expression of an HIV-1 structural protein which identified CRNKL1, ISY1, XAB2 and BUD31 as top hits [97]. CRNKL1 associated with unspliced HIV1 RNA in the nucleus and its depletion decreased splicing and HIV-1 RNA nuclear export efficiency. Transcriptomics confirmed the accumulation of unspliced HIV-1 RNA and also showed that CRNKL1 depletion affected the cytoplasmic expression levels of ~3700 transcripts and impacted ~4% of splicing events as well but no functional enrichment was found for the alternatively spliced RNAs. Altogether, more than 30 years of research have shown that the NTC and PRP19-associated complexes are essential regulators of gene expression that accompanies and processes eukaryotic mRNAs from their transcription to their export out of the nucleus.

## 7. The NTC and Associated Proteins as Guardians of Genome Stability

In human cells, a first direct link between PRP19 and the DDR came from its identification as a direct interactor of terminal deoxynucleotidyl transferase (TdT), a template-independent DNA polymerase specifically expressed in lymphoid cells undergoing V(D)J recombination [98]. An interaction was shown in vitro and in vivo between PRP19, CDC5L and the TdT BRCT (BRCA1 C-Terminus) phosphoprotein-binding domain. The same study showed that PRP19 binds double-stranded (ds) but not single-stranded (ss) DNA non-sequence specifically and its depletion led to decreased repair of IR-induced DSBs and enhanced sensitivity to MMC, etoposide and IR, demonstrating the relevance of PRP19 for tolerance of genotoxic stress. The Legerski group found that the human NTC interacts with the Werner premature aging syndrome helicase and was required for processing ICL-containing plasmids in cell-free assays and for psoralen-crosslinked plasmid reactivation in vivo, suggesting that the role of PRP19/PSO4 in interstrand crosslink repair is evolutionarily conserved across eukaryotes [99]. The same group found that PRP19 is ubiquitylated in response to damage and that this modification blocks its association with CDC5L and PLRG1 potentially altering the structure of the core complex [100]. This finding is also supported by a large scale proteomics study that identified PRP19 as being ubiquitylated in response to UV [101].

## 8. The NTC Promotes ATR Activation

**Figure 4:**
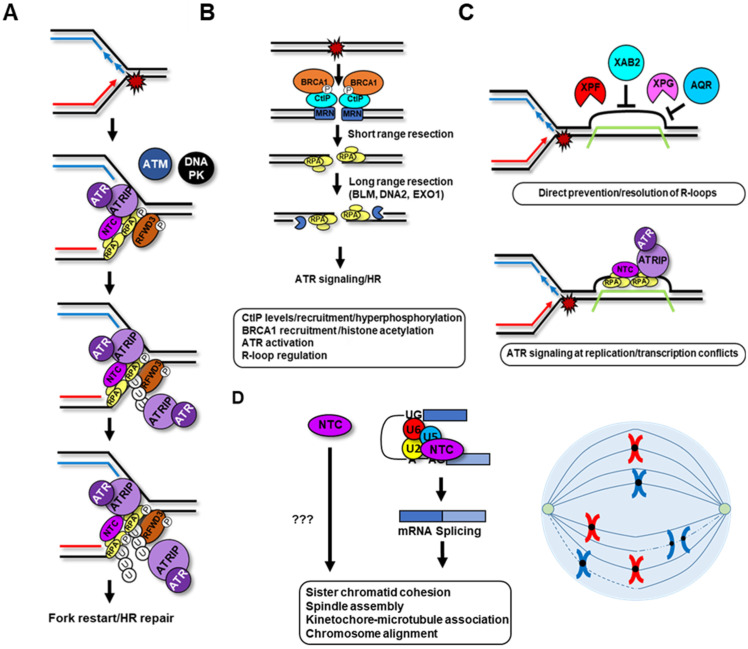
**The NTC and PRP19-associated complex proteins maintain genome stability**. (**A**) ATR activation. Fork uncoupling creates RPA-ssDNA constitutively associated with the RFWD3/FANCW ubiquitin ligase. RPA32 hyper-phosphorylation by ATR, ATM and DNA-PK enhances NTC recruitment. PRP19 and RFWD3 poly-ubiquitylate the RPA complex with various ubiquitin chain types. NTC-mediated ubiquitylation helps tether ATR-ATRIP to RPA-ssDNA creating a feed-forward loop that spreads RPA phosphorylation and ubiquitylation across RPA-ssDNA filaments which stimulates replication stress signaling, fork restart and homologous recombination. (**B**) Control of double-strand break resection. Immediately after break induction in S/G2, a phosphorylation/ubiquitylation cascade brings BRCA1 and CtIP to double-stranded DNA ends. CtIP stimulates the endonuclease activity of the MRN complex and promotes short range resection of the breaks. Long range resection factors BLM, DNA2 and EXO1 along with RPA allow production of longer ssDNA overhangs to promote ATR signaling and HR. NTC and associated factors influence CtIP levels, phosphorylation and recruitment. BRCA1 recruitment is also perturbed by downregulation of XAB2 and other splicing factors. Attenuation of ATR signaling in NTC-depleted cells could also contribute to resection defects. (**C**) R-loop prevention and mitigation. The XAB2 and AQR NTC-associated factors limit R-loop accumulation. XAB2 is also involved in recruitment of the nucleases XPF and XPG which can cleave RNA:DNA hybrids and produce replication-associated breaks. At replication–transcription conflicts, the NTC could locally activate ATR on the RPA-ssDNA portion of longer R-loops. (**D**) Mitosis regulation. The NTC complex functions directly during mitosis to promote spindle assembly and chromosome alignment during metaphase. The NTC and multiple other splicing factors are also required for mitosis by enabling the proper splicing of cohesion and mitotic gene mRNAs, particularly that of sororin.

In response to DNA replication stress or following resection of DSBs, the NTC complex relocates onto RPA-coated single-stranded DNA (RPA-ssDNA) and promotes ATR activation [37,102,103,104,105]. Depletion of PRP19, CDC5L, PLRG1 or SPF27 strongly decrease ATR recruitment and activation at stalled forks as measured by ATRIP foci formation and phosphorylation of its canonical substrates RPA and CHK1 [37,102,103,104]. Furthermore, NTC KD blocks fork restart and DSB repair by homologous recombination (HR), two important processes controlled by the ATR branch of the DDR [106,107]. Importantly, a PRP19 WD40 domain mutant that cannot interact with RPA but still forms the NTC splicing complex cannot promote ATR activation, HR and repair of broken replication forks, providing support for a dual role of PRP19 in mRNA processing and the DDR [102,106]. U-box deletion or mutation also impedes ATR activation and HR, implicating the E3 ubiquitin ligase activity of PRP19 in these processes. Mechanistically, we and others have found that the NTC interacts directly with the RPA complex via PRP19 and SPF27 and promotes its ubiquitylation [102,103]. Indeed, PRP19 or PLRG1 depletion decreases RPA70 and RPA32 ubiquitylation in response to the topoisomerase I inhibitor camptothecin (CPT) or the ribonucleotide reductase inhibitor hydroxyurea (HU) [37,102,106]. RPA ubiquitylation is mediated in part by K63-linked chains and ATRIP exhibits affinity for this type of chain which may promote ATR-ATRIP recruitment onto RPA-ssDNA [102,108]. In vivo, PRP19 works with the RFWD3/FANCW Fanconi anemia ubiquitin ligase on RPA-ssDNA and both ligases are required for maximal RPA ubiquitylation [37,102,106,108,109]. Whereas RFWD3 is constitutively associated with RPA, RPA32 hyperphosphorylation at its N-terminus by the ATR, ATM and DNA-PK kinases triggers NTC association and RPA ubiquitylation [37,102,106,108,110]. PRP19 also co-purifies with RFWD3 in response to CPT, perhaps by joining it on RPA-ssDNA as PRP19 interacts with the RPA70 N-terminal OB-fold whereas RFWD3 associates with the RPA32 C-terminal winged-helix domain [102,106,110,111,112]. Altogether, these data provide support for a model in which RPA hyper-phosphorylation enhances its interaction with PRP19 and its ubiquitylation by RFWD3 and PRP19 leading to fork restart and HR (Figure 4A), [102,106]. More recently, the SUMO protease SENP6 was also shown to associate in a SUMO-independent manner with the NTC and to regulate the SUMOylation levels of multiple DDR and cohesion factors [113]. Depletion of SENP6 also led to impaired ATR activation that correlated with decreased ATRIP on chromatin in response to aphidicolin, mimicking the impact of CDC5L depletion on the replication stress response. This further supports the idea that crosstalk between SUMOylation and ubiquitylation could regulate ATR-ATRIP localization and activation on RPA-ssDNA as suggested by other studies [114].

Interestingly, the effects of PRP19 and RFWD3 KD on the DDR present some close similarities but also some intriguing differences. For instance, depleting PRP19 or RFWD3 sensitizes cells to UV, mitomycin C, cisplatin and HU and leads to fork restart and HR defects [104,107,115,116,117]. However, RPA and CHK1 phosphorylation and thus ATR activation defects in RFWD3-depleted cells appear to be more context-specific [108,115]. Ubiquitylation of different sites or types of ubiquitin chains on RPA may explain some of the divergence between PRP19 and RFWD3 KD regarding DDR activation. Depleting ubiquitin ligases also entails a decrease in the ubiquitylation of all their substrates. In this context, the influence of PRP19 and RFWD3 on RPA and likely additional DDR-related substrates may ultimately lead to converging as well as diverging effects on the way various DNA lesions are handled. In this regard, it was recently proposed that RFWD3 interacts with and promotes PCNA ubiquitylation to regulate replication fork progression and translesion synthesis [118,119,120]. Whether PRP19 also participates in DNA damage tolerance pathways remains an open question but it is interesting that in yeast, Prp19/Pso4 promotes mitotic recombination while also participating in error-prone repair of DNA lesions [29,30,32]. Thus, in response to replication stress, the NTC transforms into a sensor of RPA-ssDNA and functions as a ubiquitin ligase to promote ATR signaling and fork repair.

## 9. Regulation of DSB Resection by NTC Complex Members

In addition to a role in ATR activation, the NTC and associated proteins were also shown to regulate DSB resection, a critical step that commits to the HR repair pathway during the S and G2 phases of the cell cycle (Figure 4B). The Stark lab showed that XAB2 promotes end resection and is important for DSB repair via HR and single-strand annealing (SSA) [121]. XAB2 KD compromises RPA32 and RAD51/BRCA1 foci formation in CPT or IR-treated cells, respectively. XAB2, PRP19 and ISY1 depletion also impaired hyper-phosphorylation of the key resection factor CtIP. Mechanistically, XAB2 depletion led to a slight reduction in DSB-induced chromatin ubiquitylation but impaired histone acetylation marks previously associated with BRCA1 and RAD51 recruitment (H4K16Ac and H4K9Ac, respectively). In line with this, work from other groups also showed that PRP19, CDC5L, PLRG1 and ISY1 depletion impairs DSB resection and HR [37,102,106,107,121]. Combined depletion experiments did not lead to enhanced defects in HR or SSA suggesting that XAB2, ISY1 and PRP19 could function together to promote DSB repair. More recently, XAB2 was also identified in an shRNA-targeted screen for genes involved in temozolomide (TMZ) resistance [122]. XAB2 depletion impaired DSB repair and fork progression in the presence of CPT suggesting a role in single-ended DSB repair. Supporting a direct implication in this process, TMZ treatment induced XAB2 foci colocalizing with RAD51 and γ-H2A.X and XAB2 also interacted with KU80. In contrast to prior studies, resection as measured by RPA32 foci formation in TMZ-treated cells was not affected by XAB2 depletion but RAD51 and KU80 foci were enhanced suggesting that XAB2 evicts KU from single-ended DSBs. XAB2 depletion in this study also did not impact RPA phosphorylation nor affect the levels of CtIP, RAD51, RPA32 or KU70 and the hyperphosphorylation of CtIP [121]. Overexpression of RAD51 or RAD52 was able to rescue the HR-defect of XAB2-depleted cells and intriguingly, a synthetic lethal relationship was found between RAD52 and XAB2 similar to that described between RAD52 and BRCA1, BRCA2 and PALB2 [123,124]. Altogether, it appears that XAB2 promotes HR via regulating the activity of the CtIP resection factor and also by promoting removal of KU to support productive RAD51 filament formation.

ZNF830, another component of the XAB2-PRP19 complex, also promotes resection and DSB repair via HR. In this case, it was shown that ATR phosphorylation promotes ZNF830 recruitment to sites of damage. Mechanistically, ZNF830 can interact with 5′ or 3′ resected dsDNA via its zinc finger domain and with CtIP via a coiled-coil domain and was suggested to promote DSB resection by enhancing CtIP recruitment at DSBs. These results contrast with those of the Stark lab as CtIP recruitment was not affected by XAB2 depletion likely indicating distinct roles for these two factors. In accordance with its role in HR, ZNF830 depletion sensitized lung cancer cells to IR, HU and CPT and also led to olaparib sensitivity in lung cancer cells and xenografts [125].

Finally, the PRP19-associated RNA helicase AQR was also shown to regulate DSB resection and HR. These defects correlated with decreased CtIP levels in AQR-depleted cells. However, decreased RAD51 foci formation in AQR-KD cells could not be complemented by re-expressing CtIP. Instead, R-loop downregulation by RNAseHI overexpression, which specifically cleaves RNA:DNA hybrids, was found to rescue HR-defects induced by AQR KD suggesting that AQR participates in DSB resection by actively removing unscheduled R-loops (see below) [126].

Indicative of a more general role for the splicing machinery in the regulation of DNA end resection and HR, depletion of the U5 snRNP core protein PRP8 or treatment with the splicing inhibitor pladienolide B also impaired HR- and SSA-mediated repair [127]. CPT-induced RPA32 chromatin accumulation, BRCA1 IRIF formation and histone acetylation marks associated with HR were also decreased upon PlaB treatment or PRP8 depletion. Differences were nevertheless observed between both situations as PlaB treatment impaired BRCA1 expression and 53BP1 foci formation which was not the case for PRP8 depletion. 53BP1 depletion also rescued SSA defects upon PRP8 KD but not in response to PlaB treatment. Thus, the intersection points between RNA maturation factors and the regulation of DSB resection and HR are numerous and complex and whether all NTC complex and associated splicing proteins function together or within independent functional modules in the regulation of resection remains to be explored.

## 10. The NTC and RNA:DNA Hybrid Regulation

RNA:DNA hybrids generated by defects in RNA maturation are important perturbators of DNA replication [5,7,11]. A general connection between splicing factors and genome stability was made early on by an siRNA-based screen performed in the Cimprich lab to identify regulators of genome stability. Splicing factors were prominent hits of this screen as their downregulation resulted in massive genome destabilization that could be complemented by RNAseHI overexpression in some cases suggesting that unscheduled R-loops were largely responsible for DNA damage induction. Although KD of NTC core factors was also found to induce genomic instability in this screen, RNAseHI overexpression did not fully rescue this phenotype supporting additional roles in the DDR for this complex. One notable exception is the NTC-associated RNA helicase AQR which proved to be critical to avoid production of deleterious R-loop accumulation (Figure 4C) [12,13,126]. Interestingly, the NER endonucleases XPF, XPG and CSB were also found to recognize unscheduled R-loops induced by splicing defects and proposed to generate single-stranded gaps that are then converted into DSBs during DNA replication. RNA:DNA hybrid processing also required XPA, TFIIH and CSB but not XPC implicating the transcription-coupled NER pathway in this process.

Another conceptual bridge was recently established between NTC-associated factor XAB2 and R-loop resolution. XAB2 is essential for NER, mRNA splicing and R-loop processing [48,128,129]. In cells, XAB2 associates with splicing factors directly to form the PRP19-XAB2 and PRP19-associated complexes and also interacts in an RNA-dependent manner with the XPF and XPG endonucleases. Transcription-blocking DNA damage induced by exposing cells to Illudin S or UV promoted the release of XAB2 from its associated snRNAs and pre-mRNAs in a DNA damage signaling-independent manner. XAB2 KD also impaired splicing and expression of genes involved in cell cycle, transcription, DNA repair and RNA processing. DNA–RNA hybrid immunoprecipitation (DRIP) analysis revealed that XAB2 associates with R-loops induced by high transcription and Illudin S. Finally, association of XPF and XPG with R-loops was destabilized in XAB2 KD cells suggesting that XAB2 associates with R-loops and promotes the activity of XPF and XPG on these structures to protect genome stability.

An open question is whether the roles of NTC-associated factors in R-loop prevention and processing are linked to their function in DSB resection and HR. Indeed, studies in fission yeast have shown that R-loop formation prevents extended resection of DSBs but at the same time, R-loop removal is important for RPA-ssDNA formation [130]. Persistent R-loops in *S. cerevisiae* also led to decreased resection in adjacent genomic regions and impaired the repair of induced DSBs in centromeric plasmids via HR [131,132]. In other situations, R-loops were shown to form around induced DSBs and proposed to promote lesion repair when RNAseH1 overexpression was found to decrease resection and repair via HR and NHEJ [133]. Similarly, R-loops also stimulate transcription-associated HR and locally transcribed RNA was recently shown to promote HR via invasion of donor DNA and formation of hybrid structures termed DR-loops [134,135,136]. Clearly, RNA:DNA hybrids influence DNA lesion processing and repair depending on their accumulation, clearance and the transcriptional status of the genomic locus at which they occur. Given the fact that the NTC relocates onto RPA-ssDNA after damage and that RPA associates with and promotes the formation of R-loops, it is tempting to speculate that upon replication–transcription conflicts PRP19 could locally promote ATR activation, repair of DNA damage and resumption of replication [102,137]. Whether and how the NTC core complex regulates R-loop accumulation and if this segues into its roles in ATR activation, DSB resection and repair via HR is a promising investigation area.

## 11. Regulation of Cell Division by mRNA Splicing Factors

There is ample evidence for both splicing-dependent and independent NTC and PRP19-associated complex functions in the regulation of cell division (Figure 4D). Firstly, a yeast genetic screen for genes involved in sister chromatid separation and segregation isolated a *prp19* mutant with impaired chromosome segregation at anaphase. The defect was attributed to deficient spindle assembly and could be complemented by expression of an intronless α-tubulin-encoding gene, suggesting that defects in pre-mRNA processing were responsible for mitosis impairment [138]. The importance of mRNA splicing factors, including the NTC, for cell division is conserved in human cells. For instance, large scale siRNA-based microscopy screens for mitosis genes identified PLRG1 and a number of NTC-associated and other splicing factors as important regulators of mitotic progression [139,140]. The NTC and other splicing factors were further shown to promote sister chromatid cohesion via splicing regulation of cohesion-promoting genes, most prominently sororin, in both G2 and mitosis [141,142,143]. Long term PRP19, PLRG1, SPF27 or CDC5L KD in human cells all lead to chromosome misalignment during prometaphase, sustained mitotic arrest and eventual cell death [144]. In this case, the mitotic defect could be traced down to impaired microtubule–kinetochore attachment accompanied by DNA damage. Moreover, CDC5L depletion led to downregulation and mis-splicing of mitotic progression genes and DDR factors suggesting that regulation of mitosis and genome stability by CDC5L is mediated at least partly by its splicing functions. More recently, the cell division-promoting role of the NTC was extended to meiosis as depletion of CDC5L caused metaphase I arrest likely due to insufficient anaphase promoting complex activity in mouse oocytes [145]. CDC5L depletion stabilized the separase-inhibitor securin which led to separase inactivation and an overabundance of chromosome arm cohesin during meiosis I. A direct association between CDC5L and securin was also found, pointing towards a direct role for the NTC in meiosis promotion. Finally, in a recent high-throughput CRISPR-based optical pooled screen, the NTC and NTC-associated components (PRP19, CDC5L, PLRG, SNW1 and BUD31) co-clustered with other mRNA splicing factors as key regulators of mitosis, confirming prior work [146]. In addition to its splicing roles, the NTC also participates directly in mitosis progression. Elegant immunodepletion and complementation experiments in mitotic Xenopus egg extracts showed that removing the NTC induces pro-metaphase arrest and chromosome misalignment supporting its direct involvement in spindle assembly [147]. SPF27 or PRP19 depletion severely compromised microtubule–kinetochore attachment and disturbed bipolar spindle formation. In contrast to NTC-depletion, splicing inhibition by spliceostatin A or SF3A1 depletion or transcription inhibition by actinomycin D treatment did not affect spindle assembly in this system, further supporting the idea that NTC plays a direct but still uncharacterized role during spindle assembly and mitosis progression. Because of its roles in DSB repair, sister-chromatid cohesion and chromosomal alignment, it is possible that NTC depletion could enhance formation of chromosomal aberrations and promote aneuploidy in cancer cells although this remains to be formally tested.

## 12. Conclusions

Ever since its first isolation as a radiation resistance factor in yeast almost 45 years ago, PRP19 has stood out as an essential eukaryotic regulator of the gene expression program via its functions in transcription and mRNA maturation but also as a component of DNA damage signaling and repair pathways and more recently as a central actor in chromosome cohesion, alignment and segregation during mitosis. The versatility of the NTC complex comes from its U-box ubiquitin ligase and WD40 substrate binding domains which allow it to decorate a wide variety of proteins with both degradative and non-degradative ubiquitin chains, influencing the activities and levels of many key regulators of genome stability and RNA processing. Deciphering the full gamut of PRP19 targets will likely shed additional mechanistic insights on its essential cellular functions and should be a rich investigation area for the future. In particular, understanding which components of the spliceosome are targeted by PRP19 and how their ubiquitylation and deubiquitylation promotes the splicing cycle will be necessary to fully grasp the spliceosomal gymnastics that produce mature mRNAs. Identifying specific NTC substrates in cells treated with genotoxic agents and synchronized in mitosis will also be instrumental to our understanding of DNA damage signaling and the transition from metaphase to anaphase during cell division.

Studying the interplay between the NTC and PRP19-associated complexes and RNA:DNA hybrid formation and dissolution is also a promising area of research as PRP19 re-localization onto RPA-ssDNA and the promotion of ATR activation may influence the local response to R-loop accumulation. Understanding the impact of the NTC on R-loop regulation and how this translates into a more stable genome will be key to understand the contributions of PRP19 to the protection of genetic information.

Finally, another intriguing aspect of the NTC is its ability to extend the lifespan of primary human cells and even of whole organisms along with their resilience towards genotoxic stress. Indeed, PRP19 overexpression in human umbilical vein endothelial cells delayed the onset of replicative senescence and led to decreased apoptosis in response to bleomycin [148]. ATM-dependent phosphorylation of PRP19 was also shown to be required for resistance to oxidative stress-induced apoptosis while also contributing to lifespan extension of human PRP19-overexpressing cells [149]. In line with these results, the Legerski group also found that PRP19 overexpression protects HeLa cells against methyl methane sulfonate-induced apoptosis [100]. A stronger link between aging and the NTC was established when it was shown that in mice, PRP19/SNEV deletion led to embryonic lethality shortly after blastocyst formation. Heterozygous mice lacked readily apparent phenotypes but PRP19−/+ mouse embryonic fibroblasts exhibited decreased in vitro proliferative potential reaching a senescent state earlier than their WT counterparts [150]. This fits well with more recent results obtained in normal human fibroblasts [91]. Ubiquitous overexpression of the drosophila PRP19 homolog also robustly enhanced the lifespan of adult female flies and their resistance towards cisplatin [151]. Altogether, these data point towards the NTC as a possible evolutionarily conserved regulator of aging but more work is required to characterize the mechanisms through which the NTC can delay the aging process [152].

The roles of PRP19 in cancer are also still the focus of ongoing investigation and conflicting results have been reported regarding pro- or anti-cancer functions of PRP19. For instance, PRP19 overexpression in tumor tissue correlated with enhanced patient survival of breast cancer patients. Overexpression of PRP19 in lung cancer cells also decreased cisplatin-induced and impaired cell migration and tumor growth [153]. Contrastingly, PRP19 expression correlated with bone marrow metastasis in neuroblastoma and was an overall adverse prognostic biomarker with a positive effect on cell migration [154]. Unanimously, however, work from many labs over the years has shown that depletion of NTC and PRP19-associated complexes is clearly detrimental to cancer cell resistance to genotoxic stress, and thus PRP19 inhibition could potentially improve the efficacy of treatment regimens by enhancing cell death and/or senescence. In line with this idea, promising recent work has shown that elevated PRP19 expression correlates with poorer prognosis in hepatocellular cancer (HCC) patients and that PRP19 depletion enhances the efficiency of irradiation/radiotherapy in HCC cell lines and tumor models [155]. Clearly, the influence of PRP19 on cancer cell growth and migration is context-dependent and further work will be required to understand the molecular mechanisms through which this E3 ligase influences oncogenesis across cancer types and stages and to determine whether modulating its activity could provide a novel and efficient synthetic lethal target in combination with standard chemotherapeutic drugs.
